# The Diagnostic Value of B-Mode Sonography in Differentiation of Malignant and Benign Tumors of the Parotid Gland

**Published:** 2016-09

**Authors:** Ali Khalife, Mehdi Bakhshaee, Behrouz Davachi, Leila Mashhadi, Kamran Khazaeni

**Affiliations:** 1 *Department of Otorhinolaryngology, Mashhad University of Medical Sciences, Mashhad, Iran.*; 2 *Sinus and Surgical Endoscopic Research Center, Mashhad University of Medical Sciences, Mashhad, Iran.*; 3 *Department of Radiology, Mashhad University of Medical Sciences, Mashhad, Iran.*; 4 *Department of Anesthesiology, Mashhad University of Medical Sciences, Mashhad, Iran.*

**Keywords:** Benign tumor, Malignant tumor, Parotid, Ultrasonography

## Abstract

**Introduction:**

Different imaging modalities are used to evaluate salivary gland diseases, including tumors. Ultrasonography (US) is the preferred method on account of its ease of use, affordability, safety profile, and good tolerance among patients. The aim of this study was to evaluate the role of US in differentiating malignant from benign parotid tumors, in the context of previous controversy in the literature on this subject.

**Materials and Methods:**

A cross-sectional study was performed in patients who presented to Qaem Medical Center with parotid masses and who were candidates for parotidectomy between June 2013 and January 2015. Patients were initially referred for a diagnostic US of the parotid. US examinations were performed and sonographic features were reported. The tumors were then classified as benign or malignanton the basis of literature descriptions of the US features of parotid tumors, and were next diagnosed pathologically. The sensitivity, specificity, positive predictive value, and negative predictive value of US for the purpose of differentiating malignant from benign tumors were then calculated.

**Results:**

Twenty-eight patients (aged 18–92 years) underwent US of parotid masses. Twenty-three tumors were diagnosed as benign and five were diagnosed as malignant. The final histopathologic examination showed 21 benign and seven malignant tumors. The sensitivity, specificity, positive predictive value, and negative predictive value of US for differentiating malignant from benign tumors were calculated as 57%, 95%, 80%, and 87%, respectively.

**Conclusion:**

US has a high specificity in differentiating between malignant and benign tumors. However, fine needle aspiration or core needle biopsy is advocated for an exact diagnosis.

## Introduction

 Various imaging modalities have been used to assess salivary gland pathologies, including plain radiography, sialography, computed tomography (CT), magnetic resonance imaging (MRI), scintigraphy, and ultrasonography (US). Plain radiography and sialography are used to evaluate sialolithiasis, although their use has decreased over recent years in favor of CT. CT is the best single modality for the evaluation of inflammatory diseases (1–3), with a high reported sensitivity in the detection of salivary tumors (4). 

 The literature also suggests that MR imaging is even more sensitive than CT in identifying tumors. US investigation of the major salivary glands has also been routinely used over the past 30 years. In Europe and Asia, US is widely accepted as the first-line imaging method for the assessment of lymph nodes and soft-tissue diseases in the head and neck, including major salivary glands (5–8). However, according to Yousem et al. (2), US is underused in most of North America, although in experienced hands it may supplant both CT and MR in the imaging of superficial salivary gland lesions. Because of technological advances and the superficial location of the major salivary glands, most regions are now accessible using high-resolution transducers (7.5 and 16 MHz). Only a small portion of the deep lobe of the parotid gland may be hidden by the acoustic shadow of the mandible (9). There have been many reports on the differential diagnosis of parotid tumors by means of B-mode US (10–14). 

The differentiation of malignant from benign tumors ultrasonographically is important because it may diminish the need for tissue sampling and help in formulating presurgical management and secure the informed consent of patients. While some healthcare professionals claim that US is a valuable adjunct to clinical examination, accurately differentiating benign from malignant lesions and diagnosing non-focal disease (15), others maintain that definitive diagnosis is usually not possible with US alone (16). Considering this controversy, the aim of this study is to evaluate whether US can differentiate between benign and malignant parotid tumors according to the sonographic features attributed to these tumors in the literature (17–20). These features are reviewed briefly below. Pleomorphic adenomas are hypoechoic, well-defined, lobulated tumors with posterior acoustic enhancement, and may contain calcifications (21,22). The lobulated shape of the tumor is the feature that is emphasized in differential diagnosis. Warthin's tumors are oval, hypoechoic, well-defined tumors that often contain multiple anechoic areas (23–26). Furthermore, Warthin's tumors tend to be hypervascula- rized, but may also contain only short vessel segments. Hemangiomas, the most common tumors in infants, may manifest as heterogeneous lesions with sinusoidal spaces and calcifications representing phleboliths (27). Lipomas are usually oval and hypoechoic with sharp margins and hyperechoic linear structures regularly distributed within the lesion in a striated or feathered pattern (28,29). Single vessel segments may only be found using color or power Doppler US (28). 

Neurogenic tumors often contain anechoic areas (22). In the case of mucoepidermoid carcinomas, the most common of the parotid malignancies, the US features depend on the histological grade of the tumor (30). Smaller, lower-grade lesions may appear well defined and not dissimilar to pleomorphic adenoma. More aggressive lesions typically have more malignant features such as irregular margins, and appear poorly defined with heterogeneous internal architecture. Use of color Doppler may help in characterization by demonstrating increased tumoral flow and peak systolic velocity (31). Cervical lymphadenopathy may or may not be associated in these cases, and this is also very well visualized by high-frequency US.

The second most common carcinoma is adenoid cystic carcinoma. Smaller lesions appear well circumscribed like that of pleomorphic adenoma, whereas larger lesions show features of malignancy on US such as poor definition, irregular shape, and heterogeneous echo pattern (32). Lymphomas have a hypoechoic echo pattern with loss of normal echogenic hilum, although a micronodular pattern and large mass may also occur (33).

## Materials and Methods

Patients who presented to Qaem Medical Center with parotid masses and who were candidates for parotidectomy between June 2013 and January 2015 were first referred for a diagnostic US of the parotid. US examinations were performed and reported by one radiologist using a Mindray DC-7sonography machine with a 7–11 MHz linear probe. The sonographic features that were studied are based on previous reports (17–20), and are presented in Table 1. Based on the US features of parotid tumors reviewed in literature, tumors were diagnosed as benign or 

malignant. The final pathologic diagnosis of the tumors was coined by two pathologists in the same institution. The sensitivity, specificity, positive predictive value, and negative predictive value of US for differentiating malignant from benign tumors were calculated using NCSS 2008.

**Table 1:** Sonographic features investigated

**Feature**	**Definition**
Presence of focal lesion	Lesion can be focal or dispersed
Shape of lesion	Lobulated, oval, or eccentric
Definition of border	Grade 0: well defined Grade1: ≤1/3 of border poorly defined Grade 2: 1/3 to 2/3 poorly defined Grade 3: ≥ 2/3 poorly defined
Echogenicity	Anechoic; Isoechoic; Hypoechoic; Hyperechoic; Mixed
Homogeneity	Grade 0: homogenous Grade 1: ≤1/3 heterogeneous Grade 2: 1/3 to 2/3 heterogeneous Grade 3: ≥ 2/3 heterogeneous
Internal structure	Solid; Cystic; Calcifications
Acoustic shadowing/enhancement	Absent; Present
Vascularity	Hypovascular ≤2 vessels Hypervascular>2 vessels inside the lesion

## Results

**Fig 1:**
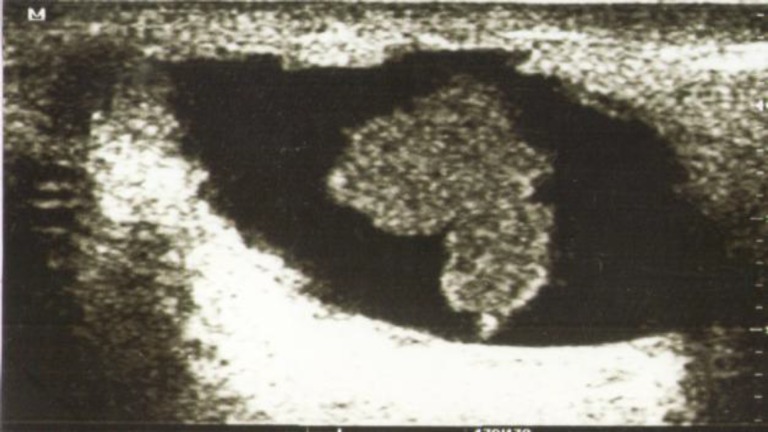
Intraductal papilloma showing dilated salivary duct with papilloma inside

Twenty-eight patients underwent US of parotid masses. The female-to-male ratio was 11:17, and ages of the participants ranged between 18 and 92 years. Depending on the sonographic features mentioned previously, 23 tumors were diagnosed as benign and five were diagnosed as malignant. The final histopathologic examination showed 21 benign and seven malignant tumors. Of the 21 benign tumors, 19 were pleomorphic adenomas and two were extremely rare cases (one myoepithelioma and one intraductal papilloma) (Fig.1).

The malignant cases included two mucoepidermoid carcinomas (one low grade and one high grade), two metastatic squamous cell carcinomas (SCC), a metastatic basal cell carcinoma (BCC), and two lymphomas (non-Hodgkin and diffuse large-cell).The histopatho- logic diagnoses as well as US classification are shown in Table.2, while the sonographic features of the masses are shown in Table.3.

**Table 2:** Histologic and pathologic diagnoses of parotid masses

**Histopathologic diagnosis**	**Ultrasonographic diagnosis** **Benign Malignant**	
Pleomorphic adenoma (19 cases)	18	1
Intraductal papilloma (1 case)	1	
Myoepithelioma (1 case)	1	
Mucoepidermoid carcinoma (2 cases)	1	1
Metastatic SCC (2 cases)	1	1
Metastatic BCC (1 case)		1
Lymphoma (2 cases)	1	1

SCC: squamous cell carcinoma, BCC: basal cell carcinoma

**Table 3:** Sonographic findings of parotid masses

	**Pleomorphic** **Adenoma**	**Intraductal papilloma**	**Myoepithel-** **ioma**	**Metastatic** **SCC**	**Muc.** **Car.**	**Metastatic** **BCC**	**Lymphoma**
**Number**	19	1	1	2	2	1	2
**Shape:**							
Lobulated	18	1		1	1		1
Oval	1		1	1		1	1
Eccentric					1		
**Distribution:**							
Focal	19	1	1	2	2	1	2
Dispersed							
**Definition of border:**							
Grade 0	16	1	1	1			2
Grade 1	1					1	
Grade 2	2			1	1		
Grade 3					1		
**Echogenicity:**							
Anechoic							
Isoechoic							
Hypoechoic	8		1	1	1		1
Hyperechoic	2						
Mixed	9	1		1	1	1	1
**Homogeneity:**							
Grade 0	4						1
Grade 1	7		1		1	1	
Grade 2	8	1		2	1		1
Grade 3							
**Internal** **Composition:**							
Solid	17			1	2	1	2
Cystic	2	1	1	1			
Calcifications	4				1		
Septations							1
**Acoustic shad- owing/enhancement:**							
Absent	1						
Enhancement	18	1	1	2	2	1	1
Shadowing							1
Mixed							
**Vascularity:**							
Hypovascular	8	1		1	1		
Hypervascular	11			1	1	1	2

**Fig 2:**
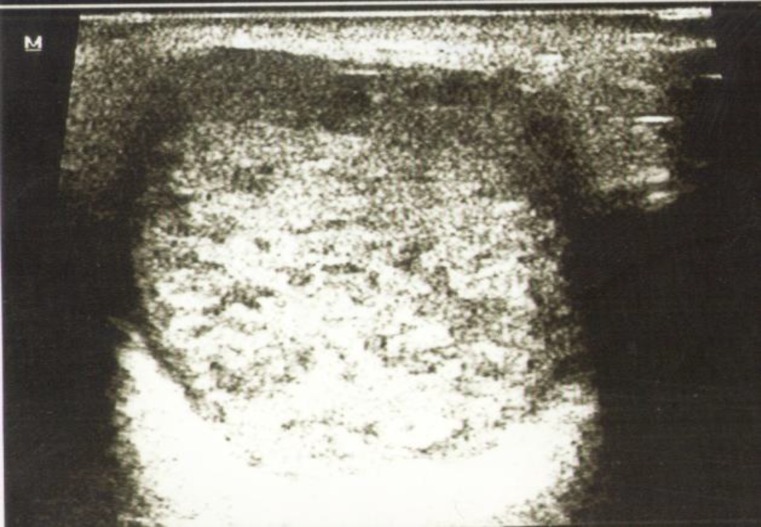
A pleomorphic adenoma showing well defined borders,hyperechoic texture and posterior enhancement

The sensitivity, specificity, positive predictive value, and negative predictive value of US for differentiating malignant from benign tumors were 57%, 95%, 80%, and 87%, respectively. Four tumors were correctly diagnosed as malignant using US, and 20 were correctly diagnosed as benign. A case of pleomorphic adenoma was falsely diagnosed as a malignant tumor and one case of mucoepidermoid carcinoma (low grade), one case of metastatic SCC, and one case of lymphoma was falsely diagnosed as a benign tumor. All of the studied masses were focal. Most of the benign tumors were pleomorphic adenomas (90%).Nearly all of the benign tumors had a lobulated shape (20 out of 21) and most of them had well-defined margins (90%). Approximately half of these tumors were hypoechoic (43%) and the other half (47%) had a mixed echotexture. Two cases were hyperechoic (Fig. 2).

**Fig 3:**
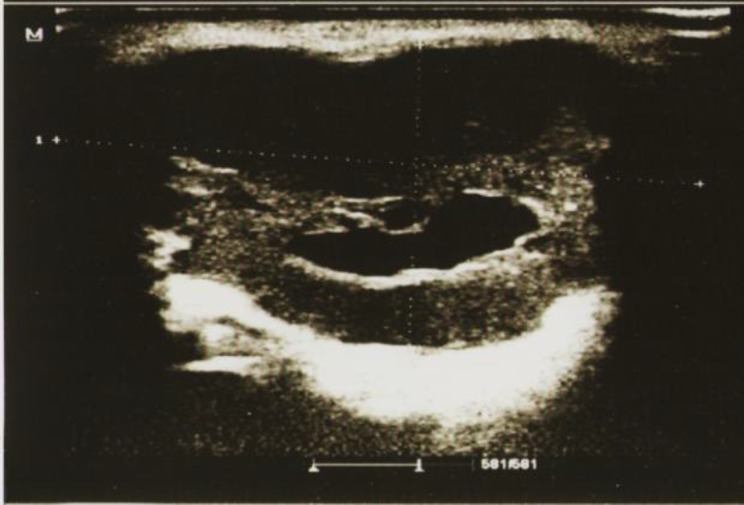
A pleomorphic adenoma showing lobulated shape, well defined margins,a cystic component and posterior enhancement

Most of the benign tumors were solid (81%) and only two were pleomorphic adenomas (Fig. 3); the intraductal papilloma and the myoepithelioma cases had cystic changes. Calcifications were seen in four out of 19 cases of pleomorphic adenomas. All benign tumors, except one case of pleomor- phic adenoma, had acoustic enhancements. Approximately half of the benign tumors were hypovascular and half were hypervascular. Fifty-seven percent of the benign tumors were homogenous (Grade 0 or 1) and 43%were heterogeneous (Grade2). 

**Fig 4:**
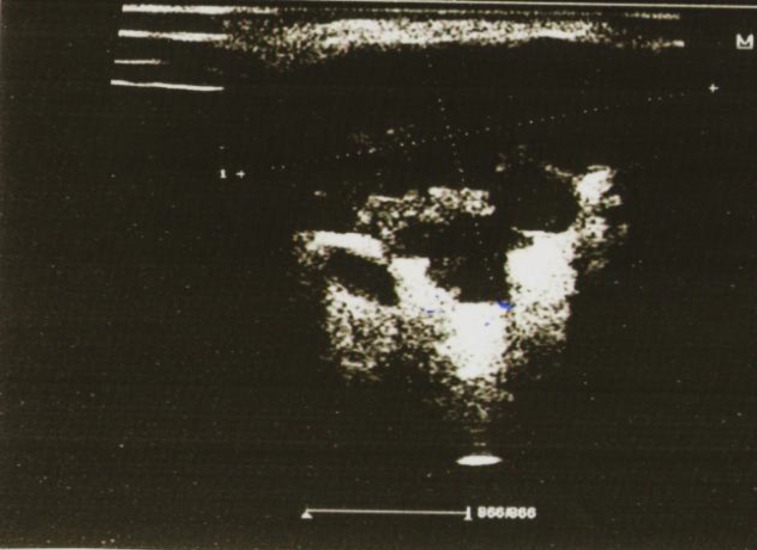
A mucoepidermoid carcinoma with eccentric shape, poorly defined margins, heterogeneous echotexture and posterior enhancement

With respect to malignant tumors, the borders were poorly defined in the two cases of mucoepidermoid carcinoma (Fig. 4), one case of metastatic SCC and the case of BCC, whereas in one case of metastatic SCC and the two cases of lymphoma they were relatively well defined. Three cases were hypoechoic and four had a mixed echotexture. Four cases were heterogeneous (Grade3) and three cases were homogeneous (Grade 0 or 1). 

All of the malignant tumors were solid except for one metastatic SCC which contained cystic foci. Only one case of mucoepidermoid carcinoma had calcifications, but all malignant tumors showed acoustic enhancements except for the diffuse large-cell lymphoma case in which internal shadowing was detected. Internal septations were observed in the non-Hodgkin lymphoma case. One case of metastatic SCC and another of mucoepider- moid carcinoma were hypovascular, but the other malignant tumors were hypervascular. The diffuse large-cell lymphoma case had a hilar pattern of vessels.

## Discussion

In this study, the majority of the benign parotid tumors were pleomorphic adenomas, which is consistent with other studies (34,35). Since only seven malignant cases were included in this study, it is not possible to identify the statistically most frequent malignant tumor. 

The sensitivity of US in differentiating malignant from benign tumors was intermediate (57%), but the specificity, positive predictive value, and negative predictive value were high, which indicates that the benign tumors were mostly correctly identified by US. The majority of pleomorphic adenomas were lobulated, had well-defined margins, and showed posterior acoustic enhancement, and a few cases had calcifications; findings that are consistent with those of Schick et al. (4,21,36).

However, in contrast to the studies previously mentioned in which pleomorphic adenomas were homogenous and hypoechoic, in our study less than half of pleomorphic adenomas exhibited these features.

Among the benign tumor cases included in this study, one was an intraductal papilloma, which is an extremely rare entity since only four cases have been reported in the parotid to date (37,38). Thus, this case merits reporting. The sonographic features of this case along with the myoepithelioma case have not been reported in the literature because of their rarity, but they were correctly diagnosed as benign due to their well-defined borders and regular shape. 

The irregular borders and heterogeneity observed in both cases of mucoepidermoid carcinoma as well as the eccentric shape of one of these tumors is consistent with the features of this tumor, as indicated in several studies (22,32,39,40).

This shows that the echotexture and border definition can be specific features of this tumor, particularly when taking into account that all benign tumors in this study had an oval or lobulated shape and relatively well-defined borders. Lymphomas, as stated in one study of Bailek et al. do not have pathognomonic features and cannot reliably be differentiated from other neoplastic lesions (41). 

However, in this same study it was mentioned that these tumors may contain echogenic septa or stripes, a finding that we observed in the non-Hodgkin lymphoma case. In the case of diffuse large-cell lymphoma, internal shadowing was detected. Miwa et al. reported that lesions with an internal shadow were malignant tumors with very high specificity (98%) (42). This finding along with the hypervascularity and hilar pattern observed in the diffuse large-cell lymphoma helped us diagnose it correctly. Regarding the metastatic SCC and BCC cases, the poorly defined borders, heterogeneity, and mixed echotexture observed in two cases of these tumors lead us to diagnose them correctly as malignant. 

The two previously mentioned features were emphasized in the paper of Howlet et al. (33), but in contrast, well-defined borders were attributed to metastasis in the work of Bialek et al. (40).

Hypervascularity was not a reliable indicator of malignancy in this study since it was observed in nearly half of the pleomorphic adenomas, although five out of seven of the malignant tumors exhibited this feature. It is stated in several papers that vascularization in pleomorphic adenomas is often poor or absent, but may be abundant (17, 20, 22). Calcification, which was noted in four cases of pleomorphic adenomas, is not a reliable indicator of malignancy since it can be observed in long-standing pleomorphic adenomas, as noted by Solbiati et al. (43).

Limitations of this study are the relatively small sample size, particularly in malignant cases, as well as the absence of color Doppler indices among the sonographic features that were investigated, which would probably have contributed to the differentiation of malignant from benign tumors.

## Conclusion

US has a high specificity in differentiating between malignant and benign tumors, and because of its simplicity, affordability, and high tolerance among patients, should be always included alongside physical examination in the diagnostic armamentarium of parotid tumors. 

However, fine needle aspiration or core needle biopsy is still advocated for an exact diagnosis. Moreover, US is a good diagnostic tool in uncooperative patients (children, the elderly, or claustrophobic patients) in whom these two tissue sampling methods, or other imaging modalities such as CT or MRI, are not feasible. 

## References

[1] Rabinov JD (2000). Imaging of salivary gland pathology. RadiolClin North Am.

[2] Yousem DM, Kraut MA, Chalian AA (2000). Major salivary gland imaging. Radiology.

[3] Silvers AR, Som PM (1998). Salivary glands. RadiolClin North Am.

[4] Bryan R, Miller R, Ferreyro R, Sessions R (1982). Computed tomography of the major salivary glands. Am J Roentgenol.

[5] Alyas F, Lewis K, Williams M (2005). Diseases of the submandibular gland as demonstrated using high resolution ultrasound. Br J Radiol.

[6] Ridder GJ, Richter B, Disko U, Sander A (2001). Gray-scale sonographic evaluation of cervical lymphadenopathy in cat-scratch disease. J Clin Ultrasound.

[7] Ying M, Ahuja A, Metreweli C (1998). Diagnostic accuracy of sonographic criteria for evaluation of cervical lymphadenopathy. J Ultrasound Med.

[8] Ying M, Ahuja A (2003). Sonography of neck lymph nodes. I. Normal lymph nodes. ClinRadiol.

[9] Katz P (1990). New applications of echography in maxillo-facial pathology. Inf Dent.

[10] Wittich GR, Scheible WF, Hajek PC (1985). Ultrasonography of the salivary glands. RadiolClin North Am.

[11] Rinast E, Gmelin E, Hollands-Thorn B (1989). Digital subtraction sialography, conventional sialography, high-resolution ultrasonography and computed tomography in the diagnosis of salivary gland diseases. Eur J Radiol.

[12] Akin I, Esmer N, Gerceker M, Aytac S, Erden I, Akan H (1991). Sialographic and ultrasonographic analyses of major salivary glands. Acta OtolaryngolStockh.

[13] Cvetinovic M, Jovic N, Mijatovic D (1991). Evaluation of ultrasound in the diagnosis of pathologic processes in the parotid gland. J Oral MaxillofacSurg.

[14] Gritzmann N (1994). Sonography of the extrathyroidal cervical soft tissues, the salivary glands and floor of the mouth. Eur J Ultrasound.

[15] Sriskandan N, Hannah A, Howlett DC (2010). A study to evaluate the accuracy of ultrasound in the diagnosis of parotid lumps and to review the sonographic features of parotid lesions - results in 220 patients. ClinRadiol.

[16] Raja V, China C, Masaki KH, Nakano G (J ClinOncol. 2002). Unusual presentations of uncommon tumors: case 1. Benign metastasizing pleomorphic adenoma.

[17] Zajkowski P, Jakubowski W, Białek EJ, Wysocki M, Osmólski A, Serafin-Król M (2000). Pleomorphic adenoma and adenolymphoma in ultrasonography. Eur J Ultrasound.

[18] Yasumoto M, Yoshimura R, Sunaba K, Shibuya H (2001). Sonographic appearances of malignant lymphoma of the salivary glands. J Clin Ultrasound.

[19] Ballerini G, Mantero M, Sbrocca M (1984). Ultrasonic pattern of parotid masses. J Clin Ultrasound.

[20] Bialek EJ, Jakubowski W, Karpinska G (2003). Role of ultrasonography in diagnosis and differentiation of pleomorphic adenomas: work in progress. Arch Otolaryngol Head Neck Surg.

[21] Schick S, Steiner E, Gahleitner A, Böhm P, Helbich T, Ba-Ssalamah A (1998). Differentiation of benign and malignant tumors of the parotid gland: value of pulsed Doppler and color Doppler sonography. EurRadiol.

[22] Shimizu M, Ussmüller J, Hartwein J, Donath K, Kinukawa N (1999). Statistical study for sonographic differential diagnosis of tumorous lesions in the parotid gland. Oral Surg Oral Med Oral Pathol Oral RadiolEndod.

[23] Shimizu M, Ussmüller J, Donath K, Yoshiura K, Ban S, Kanda S et al (1998). Sonographic analysis of recurrent parotitis in children: a comparative study with sialographic findings. Oral Surg Oral Med Oral Pathol Oral RadiolEndod.

[24] ZajkowskiP, Jakubowski W, Bialek EJ, Wysocki M, Osmólski A, Serafin-Król M (2000). Pleomorphic adenoma and adenolymphoma in ultrasonography. Eur J Ultrasound.

[25] Yu GY, Ma DQ, Zhang Y, g X, Cai ZG, Gao Y et al (2004). Multiple primary tumours of the parotid gland. Int J Oral MaxillofacSurg.

[26] Kim J, Kim EK, Park CS, Choi YS, Kim YH, Choi EC (2004). Characteristic sonographic findings of Warthin’s tumor in the parotid gland. J Clin Ultrasound.

[27] Wong KT, Ahuja AT, King AD, Yuen EH, Yu SC (2004). Vascular lesions of parotid gland in adult patients: diagnosis with high-resolution ultrasound and MRI. Br J Radiol.

[28] Gritzmann N, Rettenbacher T, Hollerweger A, Macheiner P, Hubner E (2003). Sonography of the salivary glands. EurRadiol.

[29] ChikuiT, Yonetsu K, Yoshiura K (1997). Imaging findings of lipomas in the orofacial region with CT, US, and MRI. Oral Surg Oral Med Oral Pathol Oral RadiolEndod.

[30] Joe VQ, Westessan PL (1994). Tumors of the parotid gland: MR imaging characteristics of various histological types. Am J Roentgenol.

[31] Bradley MJ, Durham LH, Lancer JM (2000). The role of colour flow Doppler in the investigation of salivary gland tumor. ClinRadiol.

[32] Howlet DC (2003). High resolution ultrasound assessment of the parotid gland. BJR.

[33] Ahuja AT, Ying M, Yuen HY, Metreweli C (2001). “Pseudocystic” appearance of non-Hodgkin’s lymphomatous nodes: an “infrequent” finding with high-resolution transducers. ClinRadiol.

[34] Lin CC, Tsai MH, Huang CC, Hua CH, Tseng HC, Huang ST (2008). Parotid tumors: a 10-year experience. Am J Otolaryngol.

[35] Takahama A Jr, Almeida OP, Kowalski LP (2009). Parotid neoplasms: analysis of 600 patients attended at a single institution. Braz J Otorhinolaryngol.

[36] Shimizu M, Ussmuller J, Hartwein J, Donath K (1999). A comparative study of sonographic and histopathologic findings of tumorous lesions in the parotid gland. Oral Surg Oral Med Oral Pathol Oral RadiolEndod.

[37] Ellis GL, Auclair PL, Ellis GL, Auclair PL, Gnepp DR (1991). Ductal papillomas. Surgical pathology of the salivary glands.

[38] Soofer SB, Tabbara S (1999). Intraductal papilloma of the salivary gland. A report of two cases with diagnosis by fine needle aspiration biopsy. ActaCytologica.

[39] Bialek EJ, Jakubowski W, Zajkowski P, Kazimierz T (2006). US of the Major Salivary Glands: Anatomy and Spatial Relationships, Pathologic Conditions, and Pitfalls. Radiographics.

[40] Howlett DC, Kesse KW, Hughes DV, Sallomi DF (2002). The role of imaging in the evaluation of parotid disease. ClinRadiol.

[41] Bradley MJ, Ahuja AT, Evans RM (2000). Salivary glands. Practical head and neck ultrasound.

[42] Miwa K, Yuasa K, Yonetsu K, Kanda S, Higuchi K, Shinohara M, Higuchi K, Kanda S, Yonetsu K, Yuasa K, Miwa K (1995). Diagnostic accuracy of ultrasonography for salivary gland tumors. Journal of the Japanese Stomatological society.

[43] Solbiati L, Osti V, Cova L, Meire H, Cosgrove D, Dewbury K, Farant P (2001). The neck. Abdominal and General Ultrasound.

